# Prognostic impact of the acute reactiveness to intravenous administration of tolvaptan sodium phosphate in patients with acute decompensated heart failure

**DOI:** 10.1093/ehjopen/oeaf108

**Published:** 2025-08-28

**Authors:** Shohei Ouchi, Hiroshi Iwata, Soshi Moriya, Ryo Naito, Norihito Takahashi, Takatoshi Kasai, Tohru Minamino

**Affiliations:** Department of Cardiovascular Biology and Medicine, Juntendo University Graduate School of Medicine, 2-1-1 Hongo, Bunkyo-ku, Tokyo, Japan; Department of Cardiovascular Biology and Medicine, Juntendo University Graduate School of Medicine, 2-1-1 Hongo, Bunkyo-ku, Tokyo, Japan; Department of Cardiovascular Biology and Medicine, Juntendo University Graduate School of Medicine, 2-1-1 Hongo, Bunkyo-ku, Tokyo, Japan; Department of Cardiovascular Biology and Medicine, Juntendo University Graduate School of Medicine, 2-1-1 Hongo, Bunkyo-ku, Tokyo, Japan; Department of Cardiovascular Biology and Medicine, Juntendo University Graduate School of Medicine, 2-1-1 Hongo, Bunkyo-ku, Tokyo, Japan; Department of Cardiovascular Biology and Medicine, Juntendo University Graduate School of Medicine, 2-1-1 Hongo, Bunkyo-ku, Tokyo, Japan; Department of Cardiovascular Biology and Medicine, Juntendo University Graduate School of Medicine, 2-1-1 Hongo, Bunkyo-ku, Tokyo, Japan

**Keywords:** Acute decompensated heart failure, Intravenous tolvaptan sodium phosphate, Diuretic resistance, Outcome

## Abstract

**Aims:**

Intravenous tolvaptan sodium phosphate (IV-tolvaptan) is a novel aquaretic agent for acute decompensated heart failure (ADHF). This study evaluated its short-term effects and prognostic implications in clinical practice.

**Methods and results:**

In this retrospective cohort of 169 consecutive ADHF patients receiving IV-tolvaptan for the first time (mean age 76.0 ± 12.7 years; 50.9% female), we measured hourly urine output over 6 h and assessed clinical and biochemical parameters at baseline and 24 h post-dose. The primary endpoint was a composite of all-cause mortality and heart failure rehospitalization. At 24 h, IV-tolvaptan significantly reduced body weight (mean difference: −1.1 ± 2.3 kg, *P* < 0.001), NT-proBNP (median change: −1704 pg/mL; *P* < 0.001), and urinary osmolality (mean change: −71.4 ± 169.4 mOsm/kg; *P* = 0.015), while raising serum sodium (mean change: 1.7 ± 2.9 mEq/L; *P* < 0.001). Six-hour urine output correlated with baseline estimated glomerular filtration rate (eGFR) (*r* = 0.34; *P* < 0.001), urinary osmolality (*r* = 0.28; *P* = 0.003), and the change in serum sodium (*r* = 0.21; *P* = 0.005). In multivariable logistic regression, renal impairment (eGFR < 60 mL/min/1.73m^2^) [odds ratio (OR) 0.2; 95% confidence interval (CI) 0.1–0.4; *P* < 0.001] and higher furosemide doses (>20 mg) (OR 0.3; 95% CI 0.2–0.6; *P* = 0.01) predicted reduced responsiveness, whereas first hospitalization (OR 2.2; 95% CI 1.1–4.5; *P* = 0.04) and high urinary osmolality (OR 2.3; 95% CI 1.0–5.4; *P* = 0.05) predicted favourable response. Kaplan–Meier analysis demonstrated a lower incidence of the primary endpoint in patients achieving ≥ 1000 mL urine output (log-rank *P* = 0.032).

**Conclusion:**

Intravenous tolvaptan sodium phosphate enhances decongestion and short-term outcomes in ADHF without worsening renal function. Early diuretic responsiveness is a robust prognostic marker.

## Introduction

Acute decompensated heart failure (ADHF) is a critical condition requiring immediate and effective treatment to relieve symptoms and improve patient outcomes. In the treatment of ADHF, managing fluid overload and congestion is the central, as these symptoms are primary drivers of hospitalization and morbidity. Diuretics play a pivotal role in the management of ADHF by promoting the excretion of excess fluid, thereby relieving symptoms and reducing cardiac workload. Based on recent guidelines,^1–4^ the management of ADHF with diuretics has undergone significant updates and new additions. While intravenous loop diuretics remain the first-line treatment for congestion in ADHF, limitations regarding high-dose loop diuretics, such as diuretic resistance, adverse electrolyte imbalances, and worsening renal function,^5,6^ necessitate the exploration of alternative or adjunctive therapies. The ADVOR trial introduced acetazolamide as a promising addition to standard loop diuretic therapy, showing that its inclusion resulted in a greater incidence of successful decongestion in ADHF patients.^7^ There is growing interest in sequential or combination diuretic therapies to improve decongestion in resistant cases, which may associate with improved outcomes of patients with ADHF.

Tolvaptan, a selective vasopressin V2 receptor antagonist, has emerged as a potential treatment option for ADHF. Tolvaptan in addition to loop diuretics has shown the efficacy in reducing congestion and body weight in ADHF patients, including the increase in free-water excretion, which leads to improved fluid balance.^8,9^ Moreover, tolvaptan also exhibits renoprotective effects, particularly in patients with chronic kidney disease and heart failure with preserved ejection fraction (EF),^10^ and it also adjusts electrolyte imbalance. However, despite these promising findings, the efficacy of tolvaptan in ADHF still remains unestablished, as tolvaptan did not contribute to an improvement in the long-term prognosis of patients with ADHF.^11,12^ Meanwhile, the Japanese guideline recommends tolvaptan as second-line therapy in adjunction with loop diuretics and it is increasingly used in the treatment of patients with ADHF in Japan.^13^ Tolvaptan sodium phosphate, a pro-drug of tolvaptan, developed for intravenous administration of tolvaptan (IV-tolvaptan), was firstly approved in Japan in March 2022 for the treatment of congestive heart failure cases who are resistant to other diuretics.^14,15^

To examine the short-term effects of IV-tolvaptan on parameters, such as urine output, body weight, electrolytes, and urine osmolality, and to assess the prognostic impact of such short-term effects of IV-tolvaptan on urine output in patients with ADHF, we conducted a retrospective observational study analysing consecutive ADHF cases who were treated with IV-tolvaptan for the first time.

## Patients and methods

### Patients, clinical manifestations, and sample collection

The present study is part of a prospective registry database that includes patients admitted to the cardiac intensive care unit at Juntendo University Hospital, Tokyo, Japan. The institutional review board approved the registry database (IRB number: H12-0871), which is publicly registered (University Medical Information Network Japan—Clinical Trials Registry, ID: UMIN000007555). The present study specifically enrolled consecutive patients who received their first administration of tolvaptan sodium phosphate (IV-tolvaptan) for the treatment of ADHF from 30 May 2022 to 27 October 2023. The study was conducted in accordance with the Declaration of Helsinki.

Data regarding background demographics, comorbidities, clinical manifestations, echocardiographic parameters, and medications were collected. The presence of pulmonary oedema, pleural effusion, and valvular disease at hospital admission was assessed by the attending physicians based on clinical symptoms and imaging findings, including chest X-ray, computed tomography (CT), and echocardiography. Blood and urine samples were obtained at the time of hospital admission for ADHF and 24 h later. Hourly urine output was measured for 6 h since the administration of IV-tolvaptan in all study participants.

### Follow-up and endpoints

Patient follow-up was performed based on the data from electrical medical record, and it was terminated at the latest time point, at which their survival was confirmed, such as the last visit date to an outpatient clinic or the last day of any hospitalization at Juntendo University Hospital. The median and the range of follow-up duration since IV-tolvaptan administration were 120 and 514 days, respectively. The primary endpoint was the composite of all-cause mortality and hospitalization for heart failure. Each component of the primary endpoint was defined as secondary endpoint.

### Statistical analysis

Continuous variables are presented as the mean ± SD for normally distributed data or median with first and third quartiles, for non-normally distributed data based on Shapiro–Wilk test. Categorical variables are presented as the actual number and frequencies (%). Quantitative data across groups were compared using the analysis of variance (ANOVA) test or the Kruskal–Wallis test. Bivariate correlations were assessed by Spearman’s non-parametric test.

Hourly urine output was compared by one-way ANOVA. Previous studies evaluating responsiveness to furosemide administration in patients with heart failure utilized following thresholds of urine output, such as 200 mL within 2 h,^16^ or 500 mL within 6 h of administration.^17^ Additionally, in the present study, 25, 50 (median), and 75 percentiles of the urine output until 6 h since IV-tolvaptan administration of entire participants were 378.8, 732.5, and 1145.0 mL, respectively. Therefore, we set the cut-off value of urine output until 6 h as 1000 mL. To see effects of IV-tolvaptan on various parameters, non-parametric Kruskal–Wallis test compared estimated glomerular filtration rate (eGFR), systolic blood pressure (BP), serum sodium level, body weight, plasma NT-proBNP level, and urine osmotic pressure before and 24 h after the administration of IV-tolvaptan. Univariate binary logistic regression analyses were conducted to explore the possible background characteristics and baseline measurements which associate with better responsiveness to IV-tolvaptan. Based on findings of univariate analyses, the adjusted model for multivariate analysis included low sodium concentration < 25 percentile (i.e. 137 mEq), 75 years old or older, male sex, initial hospitalization for heart failure, and eGFR. Moreover, to assess the correlation between different diuretic effects of IV-tolvaptan, Spearman’s non-parametric analysis evaluated the correlations between urine output, and eGFR, urine osmolality, or the change in serum sodium level. In respect of prognostic impact of the diuretic responsiveness to IV-tolvaptan in ADHF, Kaplan–Meier analysis evaluated the time to the cumulative primary and secondary endpoints followed by the log-rank test for comparisons. The prognostic impact of urine output more than 1000 mL within 6 h since administration of IV-tolvaptan was assessed using univariate and multivariate Cox proportional hazards regression analyses. Additionally, to clarify the association between urine output following IV-tolvaptan administration and the risk of primary endpoints, a cubic spline term of urine output since IV-tolvaptan was fitted in two Cox models, including (i) age and sex and (ii) age, sex, eGFR, and left ventricular EF in addition to urine output as a continuous variable to assess how the spectrum of urine output related to the hazard ratios (HRs) of the primary endpoint. Consistently, the reference of urine output in the cubic spline term was determined as 1000 mL. A *P* < 0.05 was considered to indicate statistical significance. Statistical analyses were performed using SPSS statistics version 20.0.1.0 (Armonk, NY: IBM Corp.).

## Results

### Baseline characteristics of study participants

*Table 1* shows the background demographics, complications, results of blood or urine tests, the doses of administrated furosemide, and dose of IV-tolvaptan of 169 study participants. All participants were diagnosed with ADHF. Pulmonary congestion and/or pleural effusion were present in 69.0 and 88.7% of patients, respectively. The mean age was 76.0 years, and almost the half (51.2%) was male. As cause of ADHF, valvular disease, such as regurgitation of mitral valve (43.5%), tricuspid valve (44.0%), and aortic valve (26.2%), was more frequent compared with ischaemic heart disease (14.5%). Most participants (76.3%) had impaired renal function. The mean serum sodium concentration before IV-tolvaptan administration was 139 mEq/L, and the prevalence of hyponatraemia: 135 mEq/L or less was 17.8%.

**Table 1:** Background demographics of study participants

	*n* = 169
Age, years old	76 ± 12.7
Sex, male	86, 50.9%
Body weight (kg)	58.8 ± 16.3
First time hospitalization for heart failure	75, 44.4%
Ischaemic heart disease	24, 14.2%
Pulmonary congestion^a^	117, 69.2%
Pleural effusion^a^	150, 88.8%
Left ventricular ejection fraction^b^	47.3 ± 16.8
Left ventricular ejection fraction <40%^b^	64, 37.9%
Aortic stenosis (moderate or more)^b^	9, 5.3%
Aortic valve regurgitation (moderate or more)^b^	14, 8.3%
Mitral valve regurgitation (moderate or more)^b^	42, 24.9%
Tricuspid valve regurgitation (moderate or more)^b^	36, 21.3%
Estimated right ventricular systolic pressure (eRVSP), mmHg^b^	35.2 ± 14.6
Estimated glomerular filtration rate (eGFR), mL/min/1.732	46.7 ± 30.0
Sodium (Na), mEq/L	139.3 ± 4.5
Potassium (K), mEq/L	4.2 ± 0.6
Chloride (Cl), mEq/L	104.4 ± 5.2
Blood urea nitrogen (BUN), mg/dL	35.9 ± 22.0
NT-proBNP, pg/mL	7990 (3,038, 19,251)
C-reactive protein, mg/L	0.38 (0.09–0.96)
Angiotensin converting enzyme (ACE) inhibitors	14, 8.3%
Angiotensin receptor blockers (ARB)	40, 23.7%
Angiotensin receptor neprilysin inhibitor (ARNI)	16, 9.5%
Sodium–glucose cotransporter-2 (SGLT2) inhibitors	18, 10.7%
Beta blockers	76, 45.0%
Calcium channel blockers	47, 27.8%
Loop diuretics	77, 45.6%
Oral tolvaptan	33, 19.5%
Dobutamine use	61, 36.3%
Norepinephrine use	44, 26.2%

^a^Defined by attending physicians.

^b^Defined by echocardiography.

### Administration of diuretics and urine output measurement

**Figure 1:**
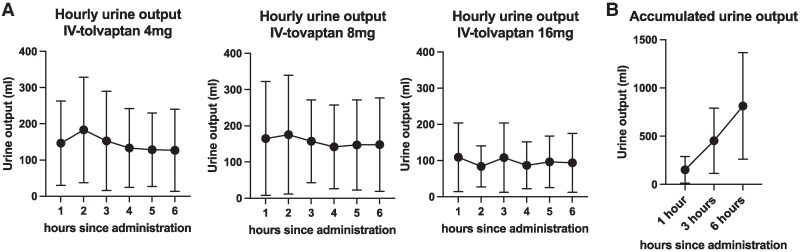
Urine output after initial administration of intravenous tolvaptan sodium phosphate. (*A*) Hourly urine output since administration of 4, 8, and 16 mg intravenous tolvaptan sodium phosphate for the first time. (*B*) Accumulated UV after tolvaptan administration and accumulated urine output at 1, 3, and 6 h since injection of intravenous tolvaptan sodium phosphate. Closed circles and error bars indicate the mean and SD of urine output at each time point, respectively.

For the treatment of ADHF, in addition to IV-tolvaptan, 87.4% of study participants received intravenous furosemide administration either as a bolus or by continuous infusion. The median time difference between the administration of furosemide and that of IV-tolvaptan (−16 to 23 h) was 2 h. The median and the range of intravenously administered furosemide dose were 20 and 0–240 mg, respectively. Moreover, as an initial dose, 53% of the study participants (*n* = 89) received 8 mg of IV-tolvaptan, and 26% (*n* = 18) and 36.3% (*n* = 61) received 4 and 16 mg, respectively (*Table 2* and *Figure 1A*). Hourly output by each dose of IV-tolvaptan is demonstrated in *Figure 1B*. In patients who received 4 and 8 mg IV-tolvaptan, urine output between 1 and 2 h since administration tended to be higher, while the difference was not statistically significant. Urine output in patients with 16 mg IV-tolvaptan was substantially lower compared with those who received 4 and 8 mg. In addition, cumulative urine output progressively increased up to 6 h in entire participants, and its mean ± SD was 813.7 ± 551.8 mL (*Figure 1B*).

**Table 2:** Intravenously administered diuretics

Dosage of furosemide (*n*, %)	
<40 mg/day	90 (53.6)
40–80 mg/day	65 (38.7)
≥ 80 mg/day	13 (7.7)
Dosage of IV-tolvaptan (*n*, %)	
4 mg	61 (36.3)
8 mg	89 (53.0)
16 mg	18 (10.7)

### Changes in parameters before and after the administration of intravenous tolvaptan sodium phosphate

**Figure 2:**
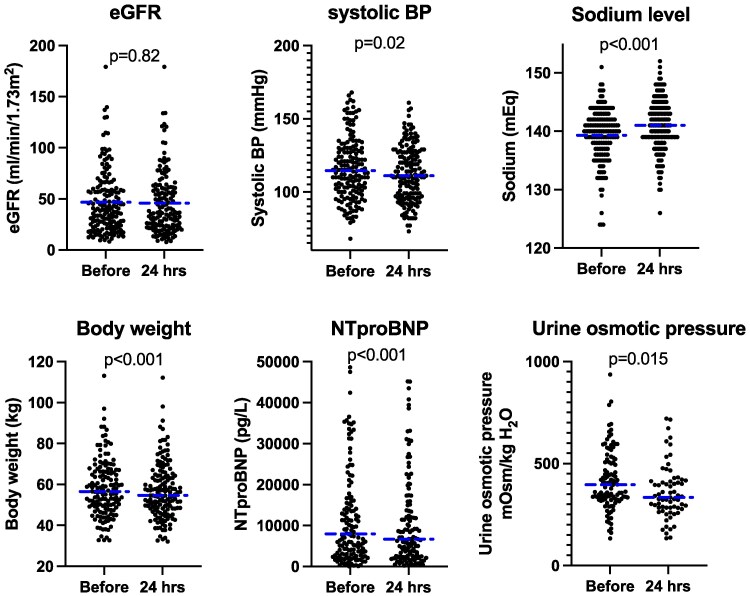
Changes in various parameters before and 24 h after the administration of intravenous tolvaptan sodium phosphate. Non-parametric Wilcoxon matched-pairs signed rank test compared parameters before and 24 h after intravenous tolvaptan sodium phosphate injection. Each circle represents level of parameters in an individual case, and blue dashed lines indicate the median value of parameter.

In addition to serum sodium concentration and urinary osmotic pressure, body weight, systolic BP, eGFR, and NTproBNP were measured and compared before and 24 h after IV-tolvaptan injection. While no significant change was observed in eGFR, body weight, NTproBNP, and systolic BP significantly decreased at 24 h after the administration of IV-tolvaptan compared with baseline levels. Moreover, IV-tolvaptan induced a significant increase in serum sodium level (139 ± 4.5–141.0 ± 4.4). However, no patient required any additional treatment for increased serum sodium level. And IV-tolvaptan induced a significant decrease in urine osmotic pressure (421.7 ± 157.0–358.33 ± 130.9) (*Figure 2*).

### Factors associated with the responsiveness to intravenous tolvaptan sodium phosphate

**Figure 3:**
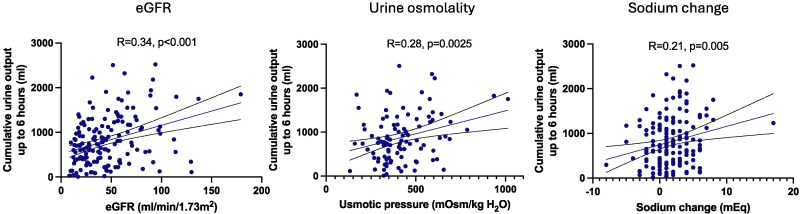
Correlations between cumulative urine output until 6 h since intravenous tolvaptan sodium phosphate injection and baseline eGFR, baseline urinary osmolality, and the change in sodium before and 24 h after intravenous tolvaptan sodium phosphate administration. Each circle represents values in individual case.

As given in *Table 3*, univariate analyses showed that the first hospitalization for heart failure, higher urine osmotic pressure (>396 mOsm/kg H_2_O), and higher eGFR were positively associated with better reactivity, while higher doses of furosemide coadministration (>20 mg) and dobutamine use were negatively associated. Time difference between the administration of furosemide and that of IV-tolvaptan was not significantly associated with better responsiveness for IV-tolvaptan administration. Moreover, a multivariate binary logistic regression analysis with urine output of 1000 mL or more within 6 h as the dependent variable and gender, age 75 years or older, initial hospitalization for heart failure, eGFR, and dobutamine use as independent variables showed a significant correlation with renal dysfunction and dobutamine use. Notably, early diuretic responsiveness to IV-tolvaptan was not defined solely by clinical indicators of right or left heart failure, as pleural effusion and pulmonary oedema did not independently predict response. We confirmed that there were no strong correlations among the independent variables. The model’s χ^2^ test was significant with *P* < 0.05, and each variable was also significant. The Hosmer–Lemeshow test had a *P*-value of 0.937, and the discriminative accuracy was 73.6% (*Table 3*). Although univariate analyses indicated that elevated urinary N-acetyl-β-D-glucosaminidase (NAG) and high urinary osmotic pressure were significantly correlated with worse and better responsiveness to IV-tolvaptan, respectively, they were not included in the multivariate model due to a relatively high number of missing data. Moreover, consistent with univariate and multivariate binary logistic regression analyses, a positive correlation was observed between the urine output within the first 6 h of administration and baseline eGFR (*r* = 0.34, *P* < 0.001). A positive correlation was also observed between the urine output and baseline urine osmolality (*r* = 0.28, *P* = 0.0025), and the difference in serum sodium levels before and 24 h after administration (*r* = 0.21, *P* = 0.005), respectively (*Figure 3*).

**Table 3:** Factors associated with good responsiveness to intravenous tolvaptan sodium phosphate

	Univariate				Multivariate			
	Odds ratio	95% CI		*P*	Odds ratio	95% CI		*P*
Na^a^ < 25 percentile (137 mMol/L)	0.5	0.2	1.0	0.06	0.5	0.2	1.3	0.15
75 years or older	1.5	0.8	2.9	0.26	1.8	0.7	4.3	0.19
Male sex	1.3	0.7	2.5	0.44	2.0	0.9	4.5	0.10
First hospitalization for heart failure	**2.2**	**1**.**1**	**4**.**5**	**0**.**04**	1.8	0.8	4.1	0.16
eGFR <60 mL/min/1.73m^2^	**0**.**2**	**0**.**1**	**0**.**4**	**<0**.**001**	**0**.**2**	**0**.**1**	**0**.**5**	**<0**.**001**
Dobutamine use	0.2	0.11	0.53	<0.001	**0**.**2**	**0**.**1**	**0**.**6**	**0**.**002**
Reduced ejection fraction (<40%)	1.9	1.0	3.8	0.05				
Mitral valve regurgitation^b^	1.7	0.8	3.9	0.18				
Pulmonary congestion	1.1	0.6	2.4	0.72				
Pleural effusion	2.5	0.7	9.2	0.16				
Oral tolvaptan	0.6	0.2	1.4	0.22				
Dose of coadministrated furosemide > 20mg	**0**.**3**	**0**.**2**	**0**.**6**	**0**.**001**				
Tricuspid regurgitation^b^	0.8	0.4	1.7	0.50				
Pulmonary hypertension^c^	1.0	0.5	2.0	0.91				
Ischaemic heart disease	0.9	0.3	2.3	0.80				
Urinary NAG^d^ > median (474.5 U/L)	0.5	0.2	1.1	0.07				
Urinary osmotic pressure > median (396 mOsm/kg H2O)	**2**.**3**	**1**.**0**	**5**.**4**	**0**.**05**				
Time difference between the administration of furosemide and that of IV-tolvaptan	1.03	0.96	1.09	0.45				

Variables with statistical significance indicated in bold.

^a^Na, serum sodium level.

^b^Moderate or more.

^c^Tricuspid regurgitation peak gradient >35 mmHg.

^d^ *N*-acetyl-β-glucosaminidase.

### Outcomes following tolvaptan sodium phosphate administration

**Figure 4:**
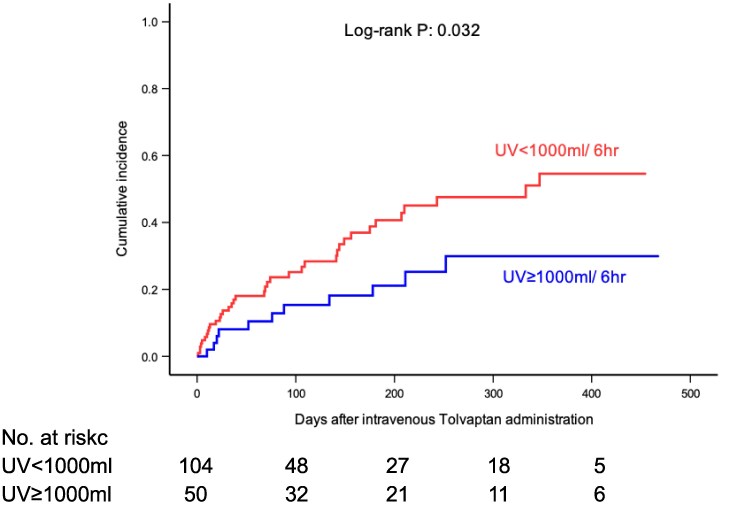
Cumulative incidences of all-cause death or heart failure hospitalization in patients with or without urine output 1000 mL or more within 6 h since intravenous tolvaptan sodium phosphate administration.

**Figure 5:**
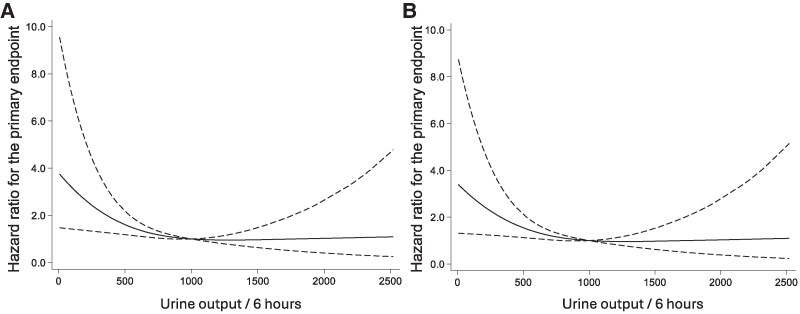
Hazard ratio and 95% confidence interval for all-cause death or heart failure hospitalization by urine output until 6 h after intravenous tolvaptan sodium phosphate administration. (*A*) Cox model was adjusted by age and sex; (B) Cox model was adjusted age, sex, Egfr, and left ventricular ejection fraction. The solid line represents the hazard ratio, and the dashed lines indicate the 95% confidence interval.

Among the 169 study participants, 37 individuals (21.9%) died, and 16 individuals (9.5%) were hospitalized for heart failure during the observation period. The composite of these events occurred in 50 individuals (29.6%). To examine the prognostic impact of the acute responsiveness to the initial administration of IV-tolvaptan, unadjusted Kaplan–Meier analyses were conducted by stratifying study patients with and without urine output 1000 mL/6 h or more following injection. Cumulative incidence of the composite of all-cause death and heart failure hospitalization was significantly lower in patients with better responsiveness (*Figure 4*). Moreover, while cumulative incidence of heart failure hospitalization was similar, the group with better responsiveness showed a significantly lower incidence of all-cause mortality (see Supplementary material online, *Figure S1*). Moreover, cubic spline curve by urine output after tolvaptan sodium phosphate injection representing age- and sex-adjusted HRs for the primary endpoint calculated by Cox regression analysis demonstrated the significant impact of the more output on the prognosis especially in patients with 1000 mL output or less (*Figure 5*). Moreover, this finding was robust even in the model adjusted by eGFR and EF in addition to age and sex.

## Discussion

In this retrospective observational cohort study of patients receiving IV-tolvaptan for the first time in routine clinical practice, we evaluated hourly urine output over the initial 6 h post-administration and assessed clinical and biochemical parameters related to IV-tolvaptan responsiveness before and 24 h after administration. The results demonstrated significant reductions in body weight, NT-proBNP levels, and urinary osmolality at 24 h post-administration, alongside a significant increase in serum sodium levels. Urine output within the first 6 h was positively correlated with baseline eGFR, baseline urinary osmolality, and the change in serum sodium levels. Unadjusted logistic regression analyses identified renal impairment and higher concurrent furosemide doses as factors associated with reduced responsiveness, while first-time hospitalization for heart failure and elevated urinary osmolality were linked to increased responsiveness. Multivariate analysis highlighted renal impairment as the sole independent factor significantly associated with responsiveness. Importantly, patients demonstrating a robust acute diuretic response, defined as urine output ≥1000 mL within 6 h of IV-tolvaptan administration, exhibited a significantly lower cumulative incidence of the primary endpoint, comprising all-cause mortality and heart failure hospitalization. Furthermore, cubic spline analysis revealed that the prognostic benefit of increased urine output was particularly pronounced in patients with urine output below 1000 mL, underscoring the prognostic importance of early diuretic response.

Diuretics, particularly loop diuretics, remain the first-line treatment for ADHF in managing congestion.^16^ Adjunctive diuretics, including thiazides or acetazolamide, may be added if congestion persists.^17,18^ Monitoring diuretic response through urine sodium content, urine output, or weight change is pivotal in the treatment of ADHF, as these metrics reflect treatment effectiveness.^19^ Moreover, emerging evidence suggests that acute improvements in right ventricular systolic function correlate with enhanced diuretic efficacy in ADHF. This relationship likely reflects relief of central venous congestion and restoration of renal perfusion pressure, thereby facilitating more effective natriuresis.^20^ While diuretics can improve symptoms and reduce mortality, they may cause electrolyte imbalances and worsen renal function.^21^ Therefore, more research is still needed to optimize diuretic strategies and their impact on outcomes.^22^ In cases of diuretic resistance, adjunctive therapies, including the addition of thiazides or acetazolamide,^18^ may be employed. The ADVOR trial demonstrated that adding acetazolamide to loop diuretic therapy improved decongestion in patients with ADHF^7^ across all renal function levels with more pronounced effects on natriuresis and diuresis in patients with lower eGFR,^23^ while it led to more frequent worsening renal function which did not correlate with adverse clinical outcomes.^23^ However, acetazolamide did not improve quality of life, shorten hospital stays, or reduce rehospitalization and death rates.^24,25^ Despite these limitations, the addition of acetazolamide represents an improvement in ADHF treatment with volume overload, though more options need consideration.^26^ Previous studies suggested that oral tolvaptan improved diuretic response, increased urine output, and relieved dyspnoea and reduced oedema and body weight more effectively than conventional treatment alone without worsening renal function in patients with ADHF.^27–29^ Moreover, early administration of oral tolvaptan preserved renal function in elderly AHF patients^30^ and may improve prognosis in responders partly by reducing the required dose of loop diuretics.^31^ Nevertheless, oral tolvaptan does not affect long-term prognosis in ADHF patients,^32^ partly because higher doses (30 mg/day) significantly worsened renal function.^29^ Overall, tolvaptan appears to be a feasible and effective adjunct diuretic therapy for ADHF patients.^33,34^ Tolvaptan sodium phosphate, a water-soluble phosphate ester pro-drug of tolvaptan, was developed for intravenous administration in patients with heart failure.^35^ Phase II and III clinical trials demonstrated that tolvaptan sodium phosphate was well tolerated and had comparable pharmacokinetics, efficacy, and safety to oral tolvaptan in patients with congestive heart failure.^14,15,36^ The development of tolvaptan sodium phosphate provides an intravenous option for individuals who have difficulty with oral intake, with expanding treatment possibilities.

In the present study, consistent with previous evidence, the administration of tolvaptan sodium phosphate to patients with ADHF resulted in increased urine output and weight loss without impacting renal function. Furthermore, its administration significantly increased blood sodium levels while decreasing urinary osmolality, which was also similar to a previous study.^37^ Among the background factors and parameters of blood or urine tests, impaired renal function was clearly associated with a poor responsiveness to tolvaptan sodium phosphate. The robust inverse association between dobutamine use and IV-tolvaptan-induced aquaresis likely reflects the presence of tissue hypoperfusion in inotrope-dependent patients, with consequent reductions in renal blood flow and glomerular filtration pressure that attenuate free-water clearance. Additionally, initial hospitalization for ADHF and high urinary osmolality were also indicated to correlate with a better diuretic response.

Similarly, predictors of better response to oral tolvaptan included preserved kidney size, higher baseline urine osmolality, and greater decrease in urine osmolality.^38–40^ However, although urine aquaporin-2 to plasma arginine vasopressin ratio was proposed as a relatively definite predictor to identify responders to oral tolvaptan,^39,40^ these parameters were not measured in this study. Baseline use of oral diuretics, including loop diuretics and oral tolvaptan, was associated with a trend towards reduced aquaretic response to IV-tolvaptan, suggesting that antecedent diuretic therapy may attenuate its efficacy. Moreover, SGLT2 inhibition and mineralocorticoid receptor antagonism may modulate the diuretic response to IV-tolvaptan, warranting evaluation in larger-scale studies. With respect to the relatively longer prognosis compared with its Phase 2 and 3 clinical trials and a preliminary study, patients with better short-term response for intravenous injection of tolvaptan sodium phosphate had significantly better outcomes than that in poorer responders. These findings are similar in a study demonstrating reduced incidence of all-cause death in patients with acute heart failure responders to oral tolvaptan.^31^ The beneficial prognostic impact of adding oral tolvaptan on the conventional diuretic therapy in patients with ADHF is not established, since a large-scale randomized trial failed to show any effect on long-term mortality in ADHF patients with reduced EF,^11^ while small-scale studies found that tolvaptan reduced the mortality rate and readmission rates in responders.^37,40^ In this study, intravenously administered tolvaptan sodium phosphate increased urine output, decreased body weight, and lowered NTproBNP levels without deteriorating renal function in addition to increasing serum sodium levels and decreasing urinary osmolality. These findings suggest that the drug exerted similar pharmacological effects with oral tolvaptan. Nevertheless, the anticipated earlier onset of effect from intravenous administration, an increase in urine volume within 1–2 h, was not evident in the present study population, as the effect was not as pronounced as with the oral formulation. Moreover, this study demonstrated that urine output within 6 h was paradoxically low in patients receiving 16 mg of tolvaptan sodium phosphate compared with those who were administered 4 and 8 mg, possibly reflecting the clinical practice of using higher diuretic doses in ADHF patients expected to have a poor response to diuretics, which may indicate real-world clinical situations not captured in in controlled research settings, such as prospective studies and clinical trials.

The most clinically relevant finding in the present study is the significant association between the short-term urine output since tolvaptan sodium phosphate administration and clinical outcomes. Although baseline renal function affected on the urine output, adjusted cubic spline curve showed the significant prognostic impact of urine output was independent from eGFR. These findings suggest that urine output was defined not only by renal function, but also by multiple factors, including haemodynamics. There is accumulating evidence that the good response for the diuretic therapy had better outcomes in the treatment of acute heart failure. Poor diuretic response is linked to advanced heart failure, renal impairment, diabetes, and atherosclerotic disease.^19^ On the contrary, good responders show a decreased risk of mortality and rehospitalization.^41^ Diuretic efficiency, a metric of responsiveness, provides prognostic information beyond fluid output or diuretic dose.^42,43^ Early treatment with tolvaptan can improve diuretic response in ADHF patients with renal dysfunction.^27^ Taken together with present and previous evidence, the responsiveness for the diuretics is a useful prognostic indicator in patients with ADHF.

Diuretic resistance remains a formidable challenge in the management of patients with acute heart failure, significantly impacting morbidity and mortality. It is characterized by a diminished natriuretic and diuretic response despite the administration of appropriate or escalating doses of diuretics. The pathophysiology of diuretic resistance is multifactorial, involving complex interactions between neurohormonal activation, renal haemodynamics, and adaptive changes in renal tubular function.^19^ Recent advancements in the treatment of diuretic resistance emphasize novel pharmacological approaches. Acetazolamide has shown efficacy in augmenting diuresis when combined with loop diuretics by promoting proximal tubular natriuresis, thereby overcoming resistance mechanisms.^7^ Sodium–glucose cotransporter 2 inhibitors like empagliflozin have demonstrated benefits in enhancing diuretic response and improving clinical outcomes in heart failure patients with diuretic resistance.^44^ Additionally, individualized diuretic strategies guided by close monitoring of diuretic response and renal function are recommended to optimize fluid management and mitigate resistance.^16^ Nesiritide, a recombinant human B-type natriuretic peptide, has been explored as a potential therapy for overcoming diuretic resistance. However, while some smaller studies suggested potential benefits in enhancing diuresis, larger trials did not show a significant advantage over placebo.^45,46^ Although the absence of a control group in this study prevents definitive conclusions regarding whether the diuretic effect would have been diminished without IV-tolvaptan, present findings demonstrate the safety and the feasibility of IV-tolvaptan to treat patients with ADHF. Moreover, we observed that patients exhibiting higher urine output following IV-tolvaptan administration, indicative of less pronounced diuretic resistance, had better subsequent prognoses. This correlation suggests the potential efficacy of IV-tolvaptan in patients with ADHF who exhibit diuretic resistance, highlighting its role as a valuable therapeutic option in this challenging clinical scenario.

### Limitations

Several limitations in the present study should be noted. First, the study included 169 patients, which may not provide a sufficiently large sample size for robust statistical analysis. Second, as a retrospective nature of the study, there is a possibility that unknown confounding factors may have influenced the results, making it challenging to fully explore the causal mechanisms behind the observed phenomena. Third, the study only examined a single group of ADHF patients who received the tolvaptan sodium phosphate, without a comparative group without that, limiting the ability to draw direct comparisons. Fourth, although the observation period was longer than in previous Phase 1 and 2 clinical trials and preliminary studies of tolvaptan sodium phosphate,^14,15,36,37^ the median duration of 130 days in the present study may still be considered insufficient for assessing long-term prognosis. Therefore, future research with larger sample sizes and longer observation periods with comparing various combinations of initial diuretic treatment is needed to provide more definitive conclusions through the present findings.

## Conclusions

Despite these limitations, the efficacy and safety of tolvaptan sodium phosphate in treating ADHF, as demonstrated in this study, are clinically significant. Furthermore, the short-term responsiveness to tolvaptan sodium phosphate has not been previously reported to be associated with prognosis, making this a novel finding that warrants further investigation in future studies.

## Lead author biography


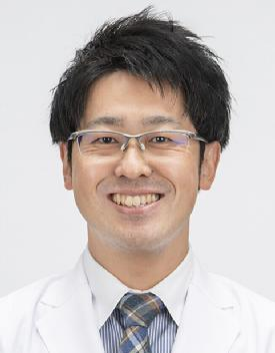


Shohei Ouchi, MD, PhD, is a cardiologist at the Cardiovascular Care Unit (CCU) of Juntendo University Hospital in Tokyo, Japan. He earned his PhD from the Graduate School of Medicine at Juntendo University. His clinical and research interests centre on interventional cardiology, with a particular focus on percutaneous coronary intervention (PCI), endovascular therapy (EVT) for peripheral artery disease, and the management of acute heart failure. He is actively engaged in cardiovascular emergency care and is dedicated to improving patient outcomes by leveraging cutting-edge medical technologies and implementing advanced, evidence-based treatment strategies.

## Supplementary Material

oeaf108_Supplementary_Data

## Data Availability

All data in this study will be available upon appropriate requests for the corresponding author, which will be assessed by primary investigator.
