# Supplementary figures and images for: Prognostic impact of the acute reactiveness to intravenous administration of tolvaptan sodium phosphate in patients with acute decompensated heart failure
Source: Eur Heart J Open. 2025 Aug 28;5(5):oeaf108. doi: 10.1093/ehjopen/oeaf108 (PMC12415332; doi:10.1093/ehjopen/oeaf108)

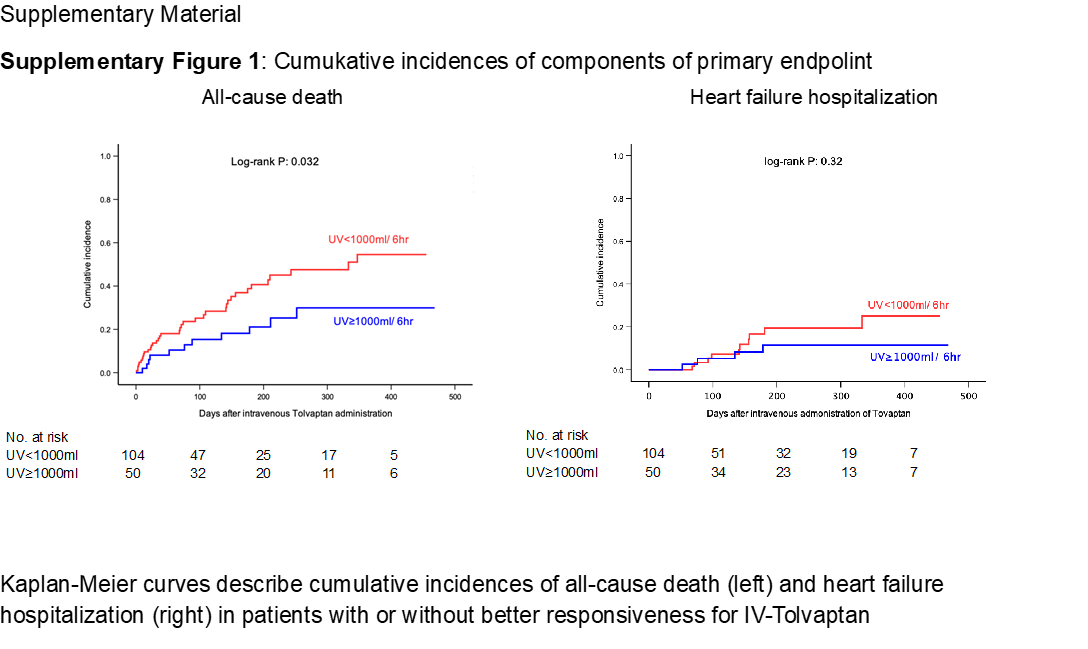

Supplement: oeaf108_Supplementary_Data [file oeaf108_supplementary_data.zip › Supplementary Material.tif]
